# Impact of AKT1 on cell invasion and radiosensitivity in a triple negative breast cancer cell line developing brain metastasis

**DOI:** 10.3389/fonc.2023.1129682

**Published:** 2023-07-06

**Authors:** Joanna Kempska, Leticia Oliveira-Ferrer, Astrid Grottke, Minyue Qi, Malik Alawi, Felix Meyer, Kerstin Borgmann, Fabienne Hamester, Kathrin Eylmann, Maila Rossberg, Daniel J. Smit, Manfred Jücker, Elena Laakmann, Isabell Witzel, Barbara Schmalfeldt, Volkmar Müller, Karen Legler

**Affiliations:** ^1^ Department of Gynecology, University Medical Center Hamburg-Eppendorf, Hamburg, Germany; ^2^ Bioinformatics Core, University Medical Center Hamburg-Eppendorf, Hamburg, Germany; ^3^ Laboratory of Radiobiology & Experimental Radio Oncology, Department of Radiotherapy and Radiation Oncology, University Medical Center Hamburg-Eppendorf, Hamburg, Germany; ^4^ Institute of Biochemistry and Signal Transduction, University Medical Center Hamburg-Eppendorf, Hamburg, Germany

**Keywords:** breast cancer, brain metastases, AKT1, radioresistance, Ipatasertib

## Abstract

**Introduction:**

The PI3K/AKT pathway is activated in 43-70% of breast cancer (BC)-patients and promotes the metastatic potential of BC cells by increasing cell proliferation, invasion and radioresistance. Therefore, AKT1-inhibition in combination with radiotherapy might be an effective treatment option for triple-negative breast cancer (TNBC)-patients with brain metastases.

**Methods:**

The impact of AKT1-knockout (AKT1_KO) and AKT-inhibition using Ipatasertib on MDA-MB-231 BR cells was assessed using in vitro cell proliferation and migration assays. AKT1-knockout in MDA-MB-231BR cells was performed using CRISPR/Cas9. The effect of AKT1-knockout on radiosensitivity of MDA-MB-231BR cell lines was determined via colony formation assays after cell irradiation. To detect genomic variants in AKT1_KO MDA-MB-231BR cells, whole-genome sequencing (WGS) was performed.

**Results:**

Pharmacological inhibition of AKT with the pan-AKT inhibitor Ipatasertib led to a significant reduction of cell viability but did not impact cell migration. Moreover, only MDA-MB-231BR cells were sensitized following Ipatasertib-treatment. Furthermore, specific AKT1-knockout in MDA-MB-231BR showed reduced cell viability in comparison to control cells, with significant effect in one of two analyzed clones. Unexpectedly, AKT1 knockout led to increased cell migration and clonogenic potential in both AKT1_KO clones. RNAseq-analysis revealed the deregulation of *CTSO*, *CYBB*, *GPR68*, *CEBPA*, *ID1*, *ID4*, *METTL15*, *PBX1* and *PTGFRN* leading to the increased cell migration, higher clonogenic survival and decreased radiosensitivity as a consequence of the AKT1 knockout in MDA-MB-231BR.

**Discussion:**

Collectively, our results demonstrate that Ipatasertib leads to radiosensitization and reduced cell proliferation of MDA-MB-231BR. AKT1-inhibition showed altered gene expression profile leading to modified cell migration, clonogenic survival and radioresistance in MDA-MB-231BR. We conclude, that AKT1-inhibition in combination with radiotherapy contribute to novel treatment strategies for breast cancer brain metastases.

## Introduction

According to cancer statistics, breast cancer (BC) is the second leading cause of cancer-related deaths in women and is the second most frequent cause of brain metastasis formation (1). Due to an increased availability and augmented quality of neuroimaging, the number of patients with diagnosed brain metastases (BM) is significantly increasing. Moreover, it is known that triple negative (TN) and HER2-positive (HER2+) types of BC have greater capacity to invade into the brain. TNBC is particularly aggressive and patients with this type have a reduced life expectancy compared to other types of BC (2). It has been shown that the median time between BC diagnosis and the appearance of BM is 35 months, whereas among TNBC patients only 21 months (3). The overall survival of breast cancer brain metastases (BCBM) patients is very poor with the median survival time 1 month when untreated and 4-6 months after local therapy and/or systemic treatment (4). Despite the improved treatment options for metastatic BC, the median survival rate of patients suffering from cerebral metastases is still worse than for other subtypes (3). For metastatic TN subtype, novel systemic therapy like the approval of checkpoint inhibitors, PARP-inhibitors and antibody drug conjugates have led mayor improvements (5).

The PI3K/AKT pathway plays a crucial role in regulating many physiological cellular functions such as proliferation and migration. However, it also represents one of the main altered pathways in multiple malignancies and is frequently hyperactivated in BC (6). A dysregulation of this pathway has been associated with treatment-resistance as well as increased angiogenesis and cellular invasion (7, 8). AKT, also known as protein kinase B, occurs in 3 isoforms: AKT1, AKT2, and AKT3. Despite the highly sequence homology of around 80%, they often have different or even opposite (patho)physiological roles (9). For instance, AKT1 reduces cell migration and metastasis formation while AKT2 promotes them (10, 11). In BC, AKT shows isoform specific effects AKT1 is mainly responsible for proliferation and survival of BC cells and has also an anti-metastatic effect. AKT2 contributes to the migration and invasion of the cells. The role of AKT3 is yet not clearly elucidated (9).

*PIK3CA/AKT1/PTEN* alterations appeared in 35% of TNBC and the frequent loss of PTEN correlates with increased AKT phosphorylation, leading to the “basal-like” BC phenotype (12). Activation of PI3K/AKT signaling pathway correlates with chemotherapy resistance suggesting to target AKT with inhibitors in combination with chemotherapeutic agents. Currently, both, pan-AKT and isoform-specific AKT-inhibitors are being evaluated for the treatment of BC (13–15). Among them, Ipatasertib, also known as GDC-0068, a selective small molecule pan-AKT inhibitor, is undergoing the second phase clinical trial, as first-line therapy for metastatic TNBC (16).

Different alterations in genes can affect the PI3K/AKT pathway activity. One of such alterations in BC is the loss of the *PTEN* gene which can lead to outgrowth of metastatic tumor cells. Hohensee et al. have shown that loss of PTEN is significantly associated with TNBC and also with shorter survival time after BM resection (17). Recent studies provide first evidence that astrocytes in the neural niche are able to downregulate the tumor suppressor PTEN in tumor cells, resulting in increased proliferation and progression of BM (18). We were able to show that PTEN overexpression in the brain seeking BC cell line MDA-MB-231BR results in decreased AKT1 kinase activity (17). Thus, confirming the finding that *PTEN* overexpression leads to a reduced AKT1 activity. In addition, it is known that AKT1 activity influences radiosensitivity of tumor cells (19). AKT protein, especially AKT1, enhances the survival among tumor cells after exposure to ionizing radiation by accelerating repair of double-strand breaks. Overall, those findings suggest that targeting AKT1 might represent a novel therapeutic target in the treatment of patients suffering from BM. Currently, GDC-0068 is undergoing clinical trials as a single agent or in combination with different systemic treatments in different tumor types (14–16). However, to date there is no data showing the impact of Ipatasertib-treatment in the context of BM treatment in BC. Here, we analyzed the functional role of AKT1 in the development of BM. Furthermore, we investigated whether the pharmacological inhibition of AKT using Ipatasertib together with radiation might have synergistic effects in the treatment of cerebral metastases.

## Materials and methods

### Cell lines and culture conditions

The TNBC cell line MDA-MB-231 was purchased by ATCC (Rockville, USA) and their brain-seeking subclone MDA-MB-231BR was a kind gift from Prof. Dr. Wikman-Kocher (Institute of Tumor Biology, University Medical Center Hamburg-Eppendorf (UKE), Hamburg, Germany). All cell lines were cultured in DMEM-medium (Gibco, ThermoFisher Scientific, Waltham, MA USA) supplemented with 10% (v/v) fetal calf serum (FCS) under standard conditions in a water-saturated atmosphere containing 5% CO_2_ at 37°C. Ipatasertib (GDC-0068) was purchased from Selleckchem (Cat.-No. S2808, Biozol, Eching, Germany, dissolved in Dimethylsulfoxide (DMSO)) and used in concentrations of 1 to 20 µM.

### CRISPR/Cas9-knockout of AKT1 in MDA-MB-231BR

AKT1 knockout (AKT_KO) in MDA-MB-231BR cell line was performed using the protocol described by Ran et al. (20). Two guide sequences of the AKT1 gene, located on exon 3 (guide #1, guide sequence: GAGCGACGTGGCTATTGTGA) and exon 4 (guide #5, guide sequence: TGGCTACAAGGAGCGGCCGC) were generated using CRISPR guide RNA design tool *guide scan*. Primers used for sgRNA oligo insert construction can be found in the **Supplementary Materials and Methods** part. The designed sgRNA oligos were used for cloning into the BbsI-cleaved pSpCas9(BB)-2A-Puro(PX459)-GFP plasmid (kindly provided by PD Dr. Stefan Werner, Institute of Tumor Biology, UKE). MDA-MB-231BR cells were seeded in 6-well-plates (3.0 x 10^5^/well) and after 24h, cells were transfected with the CRISPR/Cas9-plasmids AKT1_e3_guide#1, AKT1_e4_guide#5 or AKT1_empty vector (Ctrl) using Lipofectamine LTX (Thermo Fisher Scientific Inc., Waltham, Massachusetts, USA). Recombinant cells were passaged in DMEM-medium containing 1 µg/mL puromycin (PAA Laboratories GmbH, Pasching, Austria). The transfection efficiency was confirmed assessed by fluorescence microscopy. Transfected single cell colonies were obtained by FACS Sorting method (BD FACSAria™ III sorter, Becton Dickinson, Franklin Lakes, New Jersey, USA). The success of AKT1 knockout in MDA-MB-231BR cells was confirmed by Sanger-sequencing (Eurofins Genomics GmbH, Ebersberg, Germany). The mutations of AKT1 were analyzed using the CRISP-ID tool (21). AKT1-protein expression level was further verified using Western Blot. In the end, two AKT1_KO clones containing the e4_guide#5 (AKT1_KO_C1 and AKT_KO_G4) were expanded and used for the further *in vitro*-experiments.

### Western blot analysis

Cells were seeded in 6-well-plates (2.5 x 10^5^/well) and allowed to adhere for 24h. Whole-cell lysates were prepared using RIPA lysis-buffer containing protease inhibitor (1x, Sigma-Aldrich, St. Louis, Missouri, USA) and phosphatase inhibitor (1x, Merck Millipore, Darmstadt, Germany) and analyzed by Western blot as previously described (22). The primary antibody rabbit anti-AKT1 (#2938, Cell Signaling Technology) was used for specific AKT1-detection and the mouse anti-β-Actin (sc-47778, Santa Cruz Biotechnology) was used as loading control. Immunocomplexes were detected using peroxidase-conjugated mouse anti-rabbit IgG (sc-2357, 1:8000, Santa Cruz Biotechnology) and goat anti-mouse IgG (1030-05, 1:8000, Southern Biotech) followed by chemiluminescence detection (Westar Nova 2.0, Cyanagen Srl, Bologna, Italy) and Fuji Medical X-Ray Film (Fuji-film Corporation).

### Treatment and cell viability assays

Cells (MDA-MB-231 or MDA-MB-231BR, 5000/well) were seeded in quintuplicates in 96-well-plates, grown for 24h and treated with Ipatasertib (1, 5, 10 or 20µM) or 0.1% DMSO as control by replacing the cell culture medium for 24, 48 and 72h. MDA-MB-231BR AKT1_KO or Ctrl cells were seeded in quintuplicates as described above in cell culture serum-reduced medium and grown for 24, 48 and 72h. Cell viability was determined by Cell Proliferation Reagent WST-1 assay (Roche, Mannheim, Germany) using the manufacturer’s protocol and Microplate Absorbance Reader (Tecan Trading AG, Schweiz). Cell viability values of each cell line after 2 hours were used for normalization. For statistical analyses, each experiment was performed three-times (n=3) and results were given as mean ± S.D.

### Wound healing assay

Cells (2.3 x 10^5^/well) were seeded in duplicates in 12-well-plates and after achieving full confluency, artificial scratches were performed using a sterile 200µl micropipette tip. Cells were washed with Phosphate-Buffered Saline (PBS), containing Ca^2+^ and Mg^2+^ (+/+), and Ipatasertib (1, 2.5, 5, 10, 20 µM) was added. Images of the gaps (two images/area/well) were taken always at the same place every 6h under the light microscope (Zeiss, Oberkochen, Germany). The evaluation was performed using ImageJ Wound Healing Tool (Wayne Rasband, National Institute of Health). For statistical analyses, experiment was performed three-times (n=3) and results were given as mean ± S.D.

### Colony formation assays and radiation survival

To establish the Ipatasertib IC_20_-values of MDA-MB-231 and MDA-MB-231BR, cells were seeded in sextuplicates in 6-well-plates (250/well), grown for 24h and incubated with Ipatasertib (1-20µM) or 0.1% DMSO. After 24h, cells were washed with PBS and new cell culture medium was added for additional 10d allowing colony growth. Established colonies were fixed with 70% ethanol and stained with 1% crystal violet (Sigma-Aldrich). For IC_20_-calculation, numbers of colonies (colony≥50 cells) were counted manually under the light microscope. To analyze the effect of AKT-inhibition on radiation, cells were plated as described above and grown for 24h. Ipatasertib (IC_80_-value) or 0.1% DMSO was added followed by 2, 4, 6Gy radiation 2h later. No-radiated cells were used as control. After 24h, cell culture medium was changed and cells were incubated for additional 9 to 10d. Colonies were fixed and stained as described before, and colony growth was evaluated (23). For statistical analyses, experiment was performed two-times (n=2) and results were given as mean ± S.D.

### Transwell migration assay

Cell migration assay was performed in Boyden’s transwell chambers with 8.0µM pore size filters (Corning, NY, USA). Cells (5.000/200µL/well) were placed in triplicates in the upper side of Boyden’s chamber and were treated with 20µM Ipatasertib or 0.1% DMSO as control. In the first setting, cell suspension in cell culture medium with 5% FCS was placed in the upper side, whereas the lower chamber contained cell free culture medium with 10% FCS. In the second setting, conditioned-medium was used from astrocytes, which were previously cultured 72h in 3% FCS containing DMEM-medium. Tumor cells were cultured in conditioned-medium and were placed in the upper side of the chamber and in the lower side, cell free culture medium containing 10% FCS was added. In both setting, inserts containing cells were fixed after 24h in 4% formaldehyde and stained with 1% crystal violet. Then, cells from the upper side of the Boyden’s chamber, which didn’t migrate through the pores, were removed by wiping with a cotton swab. The stained membranes were cut out from the inserts and fixed on an object carrier using Eukitt mounting medium (Sigma-Aldrich), covered by a cover glass and assessed under the light microscope. Cells were counted manually. For statistical analyses, experiment was performed two-times (n=2) and results were given as mean ± S.D.

### Whole genome sequencing analysis

To detect genomic variants in AKT1_KO MDA-MB-231BR cells, whole-genome sequencing (WGS) was performed. AKT1_KO_C1, AKT1_KO_G4 and Ctrl cells were seeded in 6-well-plates (2.5 x 10^5^/well) and were grown to 80% cell confluency. DNA was extracted using a QIAamp DNA Mini Kit (QIAGEN, Hilden, Germany) and quantity and quality were verified by NanoDrop and gel electrophoresis with 1% agarose gel, respectively. PCR free Library construction and Sequencing (DNBseq PE150) was performed by BGI Genomics (Shenzhen, China). Further analysis of the data set can be found in **Supplementary Materials and Methods** part.

### RNA sequencing analysis

To identify differentially expressed genes (DEGs) related to AKT-dependent cell migration, invasion and radiosensitivity, RNA sequencing (RNAseq) was performed. MDA-MB-231BR AKT1_KO clone C1, clone G4 and Ctrl cells were seeded in 6-well-plates (2,5 x 10^5^/well) in quadruplicates and grown to 80% cell confluency. Cells were washed with PBS (+/+) and total RNA, treated by DNase, was isolated by using Qiagen Plasmid Midi Kit and the manufacturer’s protocol (Qiagen). Sample purity was assessed using Agilent Bioanalyzer RNA 6000 Nano Kit (Agilent Technologies Inc., CA, USA) and appropriated samples (RNA 28S/18S ≥ 1.0 and RIN ≥ 7.0) were used for transcriptome analysis. RNAseq library preparation (non-stranded mRNA enrichment method) with DNBseq PE100 sequencing were performed by BGI Genomics (Shenzhen, China). Further analysis of the data set can be found in **Supplementary Materials and Methods** part.

### Statistics

For the functional assays, statistical analyses of the data were performed with GraphPad Prism Software 8.0 (San Diego, CA, USA) and comparison among groups were made by one-way or two-way ANOVA with Bonferroni *post hoc* tests. Differences were considered statistically significant at a p-level less than 0.05.

## Results

### Impact of AKT-inhibition via Ipatasertib-treatment on MD-MB-231 BR cell proliferation and migration

**Figure 1:**
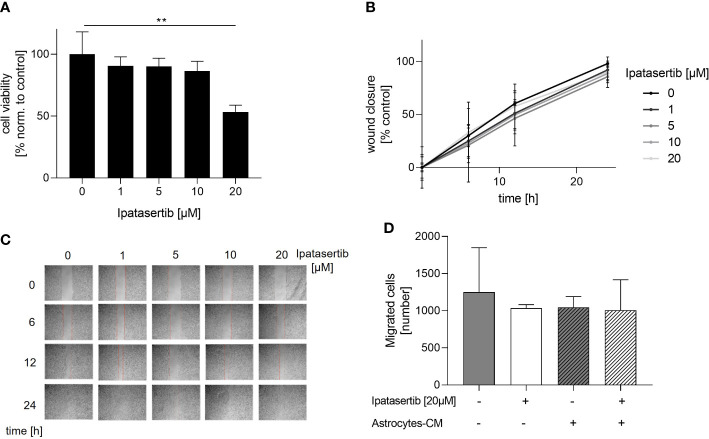
Influence of Ipatasertib on proliferation and migration of MDA-MB-231BR breast cancer cells. **(A)** Proliferation of MDA-MB-231BR cells in serum reduced growth medium after treatment with 1, 5, 10 and 20µM Ipatasertib. Cell viability was determined by WST-1 reagent after 72h. **(B)** Impact of Ipatasertib-treatment on MDA-MB-231BR cell migration ability as determined by wound healing assay. Using ImageJ Wound Healing Tool, gap closure was quantified and showed as the percentage of cleared area remaining at 6, 12 and 24h after the initial scratch. Representative results from one of three independent experiments are shown (n=3). **(C)** Representative images from one scratch assays of MDA-MB-231BR cells treated with 1, 5, 10 and 20µM Ipatasertib after 6, 12 and 24h are shown. **(D)** Influence of Ipatasertib on migration of Ipatasertib (20µM)-treated MDA-MB-231BR cells, in the absence or presence of astrocytes-conditioned-medium. Cells were placed in the upper side of Boyden`s chamber and after 24h, cell migration was detected by fixing cells on the lower side of the chamber with crystal violet staining. Data are presented as mean ± S.D. * = p<0.05; ** = p<0.01; *** = p<0.001 as assessed by one-way ANOVA with Bonferroni post hoc tests **(A)** or by two-way ANOVA with Bonferroni post hoc tests **(B)**. If not stated otherwise, p > 0.05 is considered non-significant.

To investigate the role of the serine/threonine kinase AKT1 on the cellular function of BCBM cells, we initially used the potent pan-AKT inhibitor Ipatasertib and the brain-seeking TNBC cell line MDA-MB-231BR for functional analysis. The cytotoxic effect of Ipatasertib at different concentrations (1, 5, 10, 20µM) was evaluated using the WST-1 assay. Here, Ipatasertib-treatment in a low therapeutic dose resulted in a slight reduction of the MDA-MB-231BR cell viability, whereas the higher concentration of 20µM showed a significant decrease in cell viability (**Figure 1A**). Precisely, 1µM Ipatasertib reduced the amount of vital cells to 90.50% after 72h and 20µM Ipatasertib to 53.30% (p=0.001), respectively. Besides this data, the parental cell line MDA-MB-231 showed no changes in the cell viability with the indicated Ipatasertib-concentrations. Next, we evaluated the impact of AKT-inhibition on MDA-MBA-231 BR cell migration. Therefore, we performed a wound healing-assay by scratching a cell-less area after 100% confluency of MDA-MB-231BR cells and treated them for 24h with Ipatasertib. In contrast to the cell viability assays, no change in cell migration of MDA-MB-231BR cells was observed under treatment with different Ipatasertib-concentrations after 6, 12 and 24h (**Figure 1B**). As shown in the **Figure 1C**, wounds were closed within 24h in all conditions (**Figure 1C**). To further analyze the effect of Ipatasertib on MDA-MB-231BR cell migration, Boyden`s transwell-assays were performed. AKT-inhibition resulted in no significant alteration of cell migration compared to MDA-MB-231BR untreated cells (**Figure 1D** left). In a next step, we asked whether PTEN-inhibition, via culturing the tumor cells with astrocytes-conditioned medium (astrocytes-CM), might have an impact on AKT-mediated MDA-MB-231BR cell migration and in turns on AKT-inhibition. The data demonstrated, that the astrocytes-stimulated untreated MDA-MB-231BR cells had the same values of migrated cells compared to Ipatasertib-treated MDA-MB-231BR cells (**Figure 1D** right). Furthermore, 20µM Ipatasertib-treatment had no significant impact on PTEN-mediated cell invasion compared to untreated control cells.

In conclusion, our findings indicated that the pan-AKT inhibitor, solely in high concentration, had a significant impact on the proliferation of MDA-MB-231BR cells. In contrast, AKT-inhibition had no effects on the brain seeking MDA-MB-231 cell migration, neither in the absence nor presence of astrocytes-mediated stimulating loss of PTEN.

### Effect of Ipatasertib on radiosensitization in MDA-MB-231 and MDA-MB-231BR cell lines

**Figure 2:**
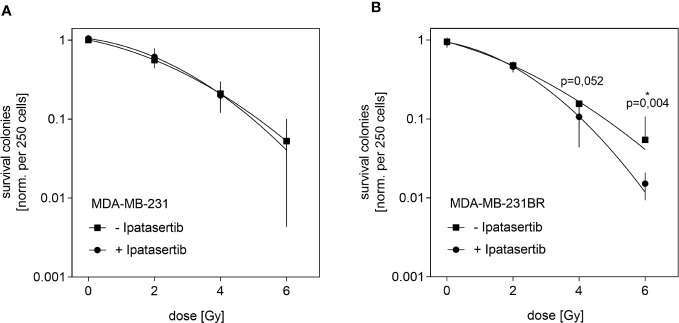
Influence of Ipatasertib on radiation in MDA-MB-231BR breast cancer cells. **(A)** MDA-MB-231 cells were treated with 2.5µM Ipatasertib and the impact on radiation was determined. **(B)** MDA-MB-231BR cells were treated with 6µM Ipatasertib and the impact on radiation was determined. Data are presented as mean ± S.D. *p<0.05 as assessed by one-way ANOVA with Bonferroni *post hoc* tests or by two-way ANOVA with Bonferroni *post hoc* tests. If not stated otherwise, p>0.05 is considered non-significant.

Next, we aimed to analyze whether the pan-AKT inhibitor Ipatasertib might sensitize BC cells for irradiation. For this purpose, we used the MDA-MB-231BR as well as the parental cell line and determined their IC_20_-value, which represents the Ipatasertib-concentration leading to 20% clonogenic survival inhibition. As a result, we obtained different IC_20_-values for both cell lines and found that the MDA-MB-231BR cell line respond more sensitively (IC_20_= 2.5µM) to Ipatasertib-treatment than the parental cell line (IC_20_= 6µM) (**Supplementary Figures 2A, B**). In the next step, both cell lines were treated with Ipatasertib for 2h using the established IC_20_-values, and subsequently irradiated with 2, 4 and 6Gy. After 10 days, cell colonies were stained with crystal violet and counted. MDA-MB-231 showed a dose-dependent reduction in cellular survival with no change after Ipatasertib-treatment (**Figure 2A**). In contrast, AKT-inhibition in the MDA-MB-231BR resulted in a significant radiation sensitization that reached statistically significant at a dose of 6Gy radiation dose (p=0.04) (**Figure 2B**).

Our results indicated, that Ipatasertib-treatment at IC_20_-value sensitize MDA-MB-231BR cells to therapeutic irradiation at higher doses.

### AKT 1 knockout in MDA-MB-231BR cells via CRISPR/Cas9-system

**Figure 3:**
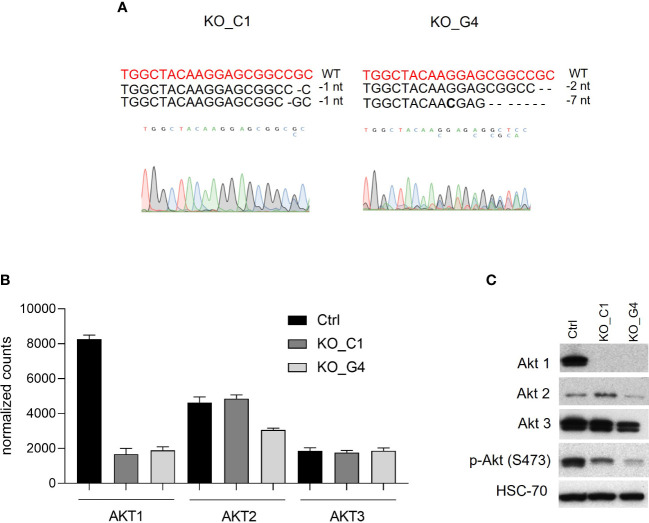
Characterization of AKT1 knockout MDA-MB-231BR breast cancer cells. **(A)** Mutation analyses was performed by using CRISP-ID tool. **(B)** Gene expression analysis of AKT1, AKT2 and AKT3 in AKT1 knockout MDA-MB-231BR cells in comparison to Ctr cells by RNAsequencing. **(C)** Expression of AKT1, AKT2, AKT3, phospho-AKT in AKT1 knockout MDA-MB-231BR cells in comparison to Ctr cells using Western blot analysis.

Since Ipatasertib is a pan-AKT inhibitor and we were interested in the specific role of the AKT1 isoform, we next established MDA-MB-231BR sublines with an AKT1 knockout. Here, two different clones, AKT1_KO_C1 and AKT1_KO_G4, and corresponding Ctrl were generated by CRISPR/Cas9-system. The Sanger-sequencing analyses revealed that in both clones the two AKT1-alleles displayed different genomic alterations and were consequently heterozygous variants (**Figure 3A**). AKT1_KO_C1 clone showed a deletion of one nucleotide on different positions at allele 1 and allele 2. The AKT1_KO_G4 clone showed on the allele 1 a deletion of two nucleotides and on allele 2 a deletion of seven nucleotides. Those results were in line with the WGS-data, which indicated, that both AKT1_KO clones were affected by frame-shift mutations and that the clone AKT1_KO_G4 showed a more pronounced somatic alteration compared to the clone AKT1_KO_C1 (**Supplementary Table 1**).

At the mRNA-level, RNAseq data showed an effective AKT1-downregulation of both AKT1_KO clones compared to Ctrl (**Figure 3B**), whereas the isoforms *AKT2* and *AKT3* were not affected. Western blot analysis confirmed the effective AKT1 knockout in MDA-MB-231BR cells at protein level.

In line with mRNA level data, AKT2 and AKT3 were not affected by AKT1 knockout as Western blot analysis revealed isoform-specific protein-bands with slight differences in expression compared to Ctrl cells. As expected, AKT1 knockout led to an effective reduced AKT-phosphorylation in MDA-MB-231BR_C1- and _G4-clones (**Figure 3C**).

Our data revealed, that effective knockout of AKT1 leading to a loss of the isoform AKT1 in both MDA-MB-231BR established clones.

### Effect of AKT 1 knockout on cell proliferation and migration in MDA-MB-231BR cells

**Figure 4:**
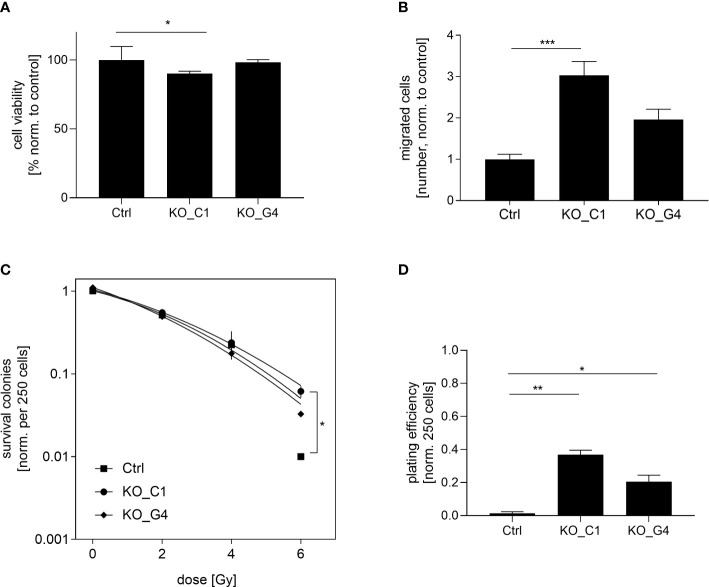
Influence of AKT1 knockout on proliferation, migration and radiation in MDA-MB-231BR breast cancer cells. **(A)** Proliferation of MDA-MB-231BR cells with AKT1 knockout (C1 clone and G4 clone) or without (Ctrl cells). Cell viability was determined by WST-1 reagent after 72h. **(B)** Influence of AKT1 knockout on migration of MDA-MB-231BR cells. Cells were placed in the upper side of Boyden`s chamber and cell migration was detected within 10d by fixing cells on the lower side of the chamber with crystal violet staining. **(C)** MDA-MB-231BR cells with AKT1 knockout or Ctrl cells were radiated with 2, 4 and 6Gy and colony formation was determined after 10d. **(D)** Colony formation ability was determined in MDA-MB-231BR cells with AKT1 knockout or AKT1 Ctrl cells. Data are presented as mean ± S.D. *p<0.05; **p<0.01; ***p<0.001 as assessed by one-way ANOVA with Bonferroni *post hoc* tests or by two-way ANOVA with Bonferroni *post hoc* tests. If not stated otherwise, p>0.05 is considered non-significant.

Next, we analyzed the functional role of AKT1 on cell proliferation. The ability of reducing cell viability was analysed in the two MDA-MB-231BR AKT1_KO clones and the values were compared to those of MDA-MB-231BR_Ctrl cells. While AKT1_KO_G4 cells behaved like the Ctrl cells, AKT1_KO_C1 cells showed a significant reduction (p=0.006) in the cell viability after 72h (**Figure 4A**). Furthermore, the impact of AKT1 knockout on tumor cell migration was evaluated using a transwell-assay with the aforementioned cell lines. Surprisingly, we observed an apparent increased cell migration in AKT1_KO clones compared to Ctrl cells (**Figure 4B**). AKT1 knockout led to faster migration of MDA-MB-231BR cells and the effect was statistically significant in AKT1_KO_C1 clone (p=0.0027) in comparison to Ctrl cells.

These results indicated that the loss of AKT1 contributes to a reduction of cell proliferation and the promotion of cell migration in MDA-MB-231BR cells.

### Effect of AKT1 knockout on radiosensitization in MDA-MB-231BR cell line

To determine the impact on AKT1-mediated radiosensitization on BC cells, AKT1_KO clones as well as the corresponding Ctrl cells were irradiated with 2, 4 and 6Gy and the clonogenic survival was analyzed as previously described. It was shown that the knockout of AKT1 led to significant radiation resistance at a dose of 6Gy compared to the Ctrl cells, which was already indicated at a dose of 4Gy (AKT1_KO_C1 p=0.01). This trend towards higher radiation resistance was also evident in the second AKT1 knockout clone (AKT1_KO_G4), but did not reach a significant level (**Figure 4C**). Next, the clonogenic potential of the aforementioned cell lines was compared in the absence of treatment. Our results showed that the colony formation ability of AKT1_KO_C1 (p=0.0195) and AKT1_KO_G4 (p=0.04) was significantly increased compared with MDA-MB-231BR_Ctrl (**Figure 4D**).

In conclusion, AKT1 knockout increases the clonogenic potential of MDA-MB-231BR cells and in turn leads to an enhanced radioresistance.

### Identification of differentially expressed genes in AKT1 knockout MDA-MB-231BR cells

To further elucidate the underlying molecular mechanism of the unexpected increased cell migration, clonogenic survival and radioresistance in the AKT1 knockout MDA-MB-231BR cell lines, especially in the AKT1_KO_C1 clone, we took a deeper look into the WGS-data and additionally performed gene expression analysis via RNAseq.

After excluding variants (SNPs and Indels), also present in the MDA-MB-231BR_Ctrl cells, we identified 118 (corresponding to 83 genes) and 123 (corresponding to 83 genes) genomic sequence variants that were unique to the AKT1_KO_C1- and AKT1_KO_G4-clone, respectively (**Supplementary Table 2**). Missense mutations were the most common mutation type in both AKT1 clones, followed by the splice site mutation, frame shift and in frame variants. Among them, those showing a deregulation at the mRNA level (RNAseq-data) and therefore with a possibly higher impact on impairing protein function were closely evaluated (**Supplementary Table 2**, bold: AKT1, GNL2, DOCK7, FLG, PLD2, ZNF236, FEM1A, LRRC75B, MAST4, EIF4G3, NBPF10, PLXNA2, MUC5B, PUS3, VPS35L, SF3B3, RACK1, HLA-A, HLA-C) regarding its potential impact on cancer cell stemness or resistance to ionizing radiation. Here, except for AKT1, no substantial association between the identified variant-associated factors and a more aggressive and stem-like phenotype or an increased sensitivity to radiation therapy in BC could be found.

**Figure 5:**
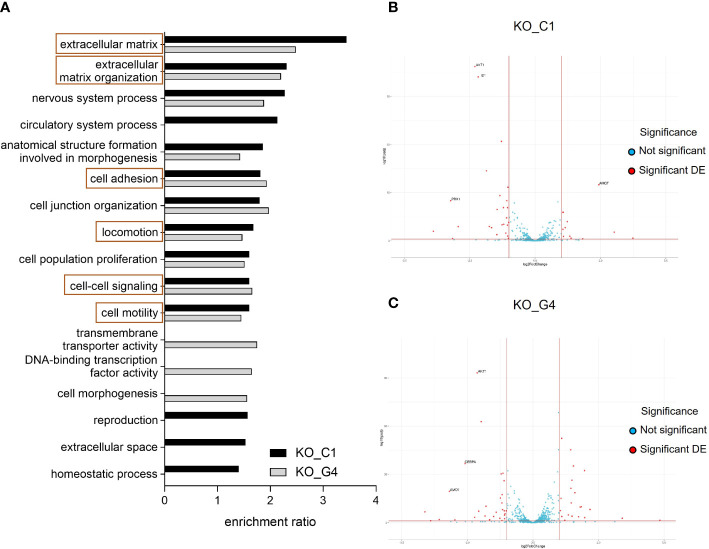
Effects of AKT1 knockout on deregulated gene expression and signaling pathway analyses in MDA-MB-231BR breast cancer cells. **(A)** Enrichment ratio of Gene Ontology terms in MDA-MB-231BR knockout cell lines in comparison to Ctrl cell line. **(B)** Volcano plot for MDA-MB-231BR AKT1_KO_C1 in comparison to Ctrl cell line. **(C)** Volcano plot for MDA-MB-231BR AKT1_KO_G4 in comparison to Ctrl cell line.

For the transcriptome comparison between MDA-MB-231BR_Ctrl, AKT1_KO_C1 and AKT1_KO_G4, DEG were mapped to cellular pathways from the Gene Ontology database. Depicted in **Figure 5**, the enrichment ratio is presented for both AKT1_KO cell lines in comparison to Ctrl (**Figure 5A**). 14 deregulated pathways in AKT1_KO_C1 and 13 deregulated pathways in AKT1_KO_G4 compared to Ctrl were found. Among them, 10 pathways were commonly deregulated in both knockout AKT1 clones, including extracellular matrix, extracellular matrix organization, cell motility and locomotion.

To have a closer look at the molecular mechanism of the increased cell migration, clonogenic survival as well as radioresistance in the MDA-MB-231BR AKT1_KO cell lines, gene sets related to stem cell differentiation, stem cell proliferation, stem cell division and radiosensitization were appropriated for the detection of deregulated genes to explain our unexpected data, especially in AKT1_KO_C1. As represented in a volcano plot, in the AKT1_KO_C1, 36 deregulated genes were identified, 11 of which were up-regulated, and 25 genes were down-regulated (**Figure 5B**; **Supplementary Table 3**). In AKT1_KO_G4, we discovered 50 deregulated genes, 21 of which genes were up-regulated and 29 genes of which were down-regulated (**Figure 5C**; **Supplementary Table 3**). Among them, 11 genes were deregulated in both AKT1 knockout clones compared to Ctrl (**Supplementary Table 3**, bold). In brief, the 10 genes *AKT1*, *CEBPA*, *CTSO*, *CYBB*, GPR68, *ID1*, *ID4*, *METTL15*, *PBX1* and *PTGFRN* were concordantly significantly deregulated in AKT1_KO_C1 and AKT1_KO_G4, whereas the gene *AMOT* was up-regulated in the clone C1 (log2Foldchange=2.436, p-value=2.569x10^-38^) and down-regulated in the clone G4 (log2Foldchange=-3.179, p-value=4.126x10^-22^). These data indicate a significantly altered gene expression profile in the two AKT knockout cell lines and could explain their aggressive and stem cell-like phenotype in comparison to MDA-MB-231BR_Ctrl cell line.

## Discussion

Over the past three decades, numerous studies have revealed a central role of the serine/threonine kinase Akt in cellular functions including cell survival, proliferation and growth (24). In BC, the PI3K/AKT pathway is activated in 43-70% of the patients with BM and the role of AKT-inhibition in BC dissemination and metastasis has been studied before (25, 26). Due to the fact that this deregulation occurs in Hormone receptor positive (HR+) disease, HER2-amplified and TN tumors, targeting the key components of the PI3K/AKT pathways seem a reasonable option for the treatment of all BC subtypes (27). Indeed, PI3K- and mTOR-inhibitors are already approved for the treatment of advanced HR+ BC patients (28, 29). In this context, AKT represents a novel pharmacological target and particularly the novel pan-AKT inhibitor Ipatasertib has already entered clinical phase III trials in HR+ and TNBC (15). However, many questions are still unresolved and our finding regarding pan-AKT inhibition and isoform-specific deficiency of AKT1 result in divergent phenotypes is highly relevant in the context of AKT-specific drug development.

AKT-inhibition resulted in slightly reduced brain-seeking MDA-MB-231 cell proliferation and this effect could be confirmed in our study. Indeed, TNBC cell lines with PIK3CA-Wt, including the parental MDA-MB-231 cells, showed moderate response to Ipatasertib (30–32). These findings were confirmed in MDA-MB-231BR cells with stable AKT1 knockout, but only in one clone (AKT1_KO_C1). Lin et al. described, that the substantial conformational change upon activation is dependent on whether the AKT protein is active, because ATP-competitive inhibitors mimicked ATP by targeting active AKT in comparison to allosteric AKT-inhibitors (33, 34).

The fact, that increased phospho-AKT activated downstream targets via phosphorylation of FoxO1 and FoxO3 leading to inhibition of cell migration (33), was not in line with our current results, showing, that AKT-inhibition did not affect MDA-MB-231BR cell migration, which exhibit diverse biological properties. Kinase assays for individual AKT isoforms revealed, that Ipatasertib showed high level of homology in the ATP-binding pockets among Akt (33). Mundi et al. reported that this strong biological activity of Ipatasertib was induced by a dose-dependent increase in AKT-phosphorylation on both residues, the kinase domain (Thr308 for AKT1) as well as on the regulatory domain (Ser473 for AKT1) in cell lines with activated PI3K/AKT pathway and these strong biological effects could also be confirmed for other ATP-competitive AKT-inhibitors (35–37). In line with published data, AKT1 knockout led to an effective reduction of AKT1-phosphorylation on Ser473 in both tested clones, whereas AKT2 was not affected in the TN brain-seeking MDA-MB-231 cell line. Interestingly, sensitivity to Ipatasertib was strongly associated with pAKT levels (pAKT S473) and tumor cell lines with *PTEN* loss, either on its protein expression or genetic mutations in *PTEN*, were significantly more sensitive to this pan-AKT inhibitor (33). Of note, our findings are based on the established pan-AKT inhibitor Ipatasertib. Although Ipatasertib is a well-studied inhibitor, that has been extensively studied in many cell lines and has no documented off-target effects on cell viability, one cannot fully exclude other off-target effects related to this molecule (38, 39).

Surprisingly, AKT-inhibition has no impact on MDA-MB-231BR cell migration but knockout of the AKT1 isoform led to a substantial increase in MDA-MB-231BR migratory potential in both AKT1 clones and more pronounced in the clone MDA-MB-231 AKT1_KO_C1. Zhang et al. found out that astrocyte-derived exosomal microRNA plays an important role during BM outgrowth (18). Other studies confirmed the findings that miRNA’s regulate the cell sensitivity through PTEN/PI3K/AKT-signaling pathway (40, 41).

Our current study demonstrates that AKT-inhibition via Ipatasertib effectively sensitizes brain-seeking TN cells to radiation. Surprisingly, the specific AKT1 knockout showed opposite results compared to the aforementioned cell line, which showed an unexpected enhanced clonogenic potential and in turn higher radioresistance. Our data are in line with Kim et al., who showed that AKT-signaling pathway blockade with the AKT-inhibitor MK-2206 significantly enhanced radiosensitivity in radioresistant cancer cell lines. These facts imply that the AKT signaling pathway could serve as a potential therapeutic target regarding radioresistance (42). One explanation for the AKT-mediated radioresistance is the activation of the DNA-dependent protein kinase catalytic subunit (DNA-PKcs) leading to DNA-double-strand break repair and to a decreased degradation of cyclin D1 as a crucial marker for cell cycle progression (43). In preclinical models, the combination of radiotherapy and AKT-inhibitors has been tested with the results that AKT-inhibitors showed radiosensitizing effects (44, 45). However, combination of AKT-inhibitors with radiotherapy have been rarely tested in clinical studies so far.

In contrast to the described role of the PI3K/AKT pathway promoting radioresistance in BC cells, our results show that particularly the AKT1 isoform rather decreases radiosensitivity, and its loss leads to an enhanced radioresistant phenotype. These results suggest that other AKT-isoforms might be involved in the Ipatasertib-mediated radiosensitization.

In recent studies, AKT isoforms were evaluated in BC and other tumors for essential activation or inhibition of its targets (9). These three different isoforms AKT1, AKT2 and AKT3 have non-redundant and partly opposing effects in tumorigenesis in BC, making inhibition with pan-AKT inhibitors inappropriate. In the context of brain metastasis in breast cancer, a previous study by our group showed that overexpression of PTEN in the brain-seeking cell line MDA-MB-231BR reduced tumor cell migration, via suppression of specifically AKT1 activity (17). Here, MDA-MB-231BR cells as well as the parental cell line MDA-MB-231 express all three AKT isoforms i.e. Akt1, Akt2 and Akt3, however an increased Akt1 activity, but not Akt2 or Akt3 activity, could be measured in the brain seeking subline in comparison with the parental one. We thus hypothesized that the effect of Ipatasertib in MDA-MB-231BR cells might be dependent on AKT1. Therefore, the role of AKT1 was analyzed in the present study with the result that two generated AKT1 knockout clones showed mainly comparable effects regarding cell proliferation, migration and radiosensitivity. Various studies showed AKT1-mediated migration and metastases formation in BC and these findings were in line with our present data (17, 46). Both generated AKT1 knockout clones are heterozygous variants and our WGS analysis showed different genomic alterations most probably leading to translational modification or deregulation of certain proteins. However, none of the affected genes could be linked with the increased stemness potential or radioresistance observed in our *in vitro*-analysis. Consequently, we can exclude that the phenotypical alterations observed in both MDA-MB-231BR knockout cell lines compared to their Ctrl cell line have arisen by clonal selection during the generation of the cell lines.

Interestingly, among 11 commonly DEG, several referred to play an important role regarding the regulation of PI3K/AKT signaling pathway. The deregulation of *CEBPA*, CCAAT enhancer binding protein α, could explain the more invasive and stem cell-like properties of the AKT1 knockout cell lines and Chang et al. depicted for the first time, that *CEBPA* is involved in chemotherapeutic resistance and cancer stemness by affecting the MAPK14/C/EBPα signaling pathway in BC (47). Another study identified *CEBPA* as a meaningful somatic mutation in altered PI3K/AKT signaling pathway in BC (48). Our present work identified *ID1*, inhibitor of DNA binding 1, HLH protein, was significantly down-regulated in both MDA-MB-231BR AKT1_KO cell lines and in line with this, the regulation of AKT-signaling by affecting ID1 was reported earlier in acute myeloid leukemia (49). In agreement with a study in which the PI3K pathway regulator *PBX1* (PBX homeobox 1) is required in BC, our findings highlight the importance of PBX1 as a genetic regulator for stemness and radioresistance in cancer (50).

## Conclusion

In conclusion, the downstream pathway of AKT1-inhibition regulates gene transcription and induced migration and clonogenic survival leading to radioresistance and our results indicate that *AKT1-*deficiency lead in a different cellular phenotype and could even promote a malignant phenotype. This data is highly relevant in the context of AKT-specific drug development and future research will show, whether Ipatasertib in combination with chemotherapy will enter the clinic for the therapeutic treatment of BCBM patients.

## Data availability statement

The datasets presented in this study can be found in online repositories. The names of the repository/repositories and accession number(s) can be found below: PRJEB55989 (ENA).

## Author contributions

Conceptualization, LO-F, AG, VM and KL; Formal analysis, JK, LO-F, FM and KL; Funding acquisition, BS and VM; Methodology, JK, FH, KE, MR and KL; Project administration, LO-F and VM; Resources, BS and VM; Software, MQ and MA; Supervision, LO-F and VM; Validation, JK, LO-F, MQ, MA, FM, FH and KL; Writing – original draft, JK, LO-F, VM and KL; Writing – review & editing, LO-F, MQ, MA, KB, FH, DS, MJ, EL, IW, BS, VM and KL. All authors contributed to the article and approved the submitted version.

## Supplementary material

Supplementary Figure 1 Influence of Ipatasertib on proliferation of MDA-MB-231 breast cancer cells. Proliferation of MDA-MB-231 cells in serum reduced growth medium after treatment with 1, 5, 10 and 20µM Ipatasertib. Cell viability was determined by WST-1 reagent after 72h. Data are presented as mean ± S.D.. Statistic was assessed by one-way ANOVA with Bonferroni post hoc tests with the result that p > 0.05 is considered as non-significant.

Supplementary Figure 2 Influence of Ipatasertib on radiation in MDA-MB-231BR breast cancer cells. **(A)** MDA-MB-231 cells were treated with Ipatasertib and were radiated with 2, 4 and 6Gy. To estimate IC_20_-value, colony formation was determined after 10d. **(B)** MDA-MB-231BR cells were treated with Ipatasertib and were radiated with 2, 4 and 6Gy. To estimate IC_20_-value, colony formation was determined after 10d. Data are presented as mean ± S.D. * = p<0.05; ** = p<0.01; *** = p<0.001 as assessed by one-way ANOVA with Bonferroni *post hoc* tests or by two-way ANOVA with Bonferroni *post hoc* tests. If not stated otherwise, p>0.05 is considered non-significant.

Supplementary Table 1 Genetic alteration in AKT1 knockout MDA-MB-231BR breast cancer cell lines using Whole-genome sequencing (WGS). Both MDA-MB-231BR AKT1_KO clones (AKT1_e4_#5_C1 and AKT1_e4_#5_G4) were affected by frame-shift mutations. The clone AKT1_KO_G4 showed a more pronounced somatic alteration compared to the clone AKT1_KO_C1.

Supplementary Table 2 Analysis of genomic sequence variants in AKT1 knockout MDA-MB-231BR breast cancer cell lines. Genomic sequence variants were identified after excluding variants (SNPs and Indels) in AKT1_KO clones and the corresponding MDA-MB-231BR Ctrl cells. 118 (corresponding to 83 genes) and 123 (corresponding to 83 genes) genomic variants were found in AKT1_KO_C1- and AKT1_KO_G4-clone, respectively.

Supplementary Table 3 Deregulated genes in AKT1 knockout MDA-MB-231BR breast cancer cell lines related to stemness- and radioresistance-pathway. 36- and 50 deregulated genes were identified in the AKT1 knockout clones C1 (up-regulated 11 genes and down-regulated 25 genes) and G4 (up-regulated 21 genes and down-regulated 29 genes), respectively. 11 genes (*AKT1*, *CEBPA*, *CTSO*, *CYBB*, GPR68, *ID1*, *ID4*, *METTL15*, *PBX1* and *PTGFRN*) were deregulated in both AKT1 knockout clones compared to Ctrl cells.

## References

[1] Bray F Ferlay J Soerjomataram I Siegel RL Torre LA Jemal A. Global cancer statistics 2018: GLOBOCAN estimates of incidence and mortality worldwide for 36 cancers in 185 countries. CA: Cancer J Clin (2018) 68:394–424. doi: 10.3322/caac.21492 30207593

[2] Witzel I Oliveira-Ferrer L Pantel K Muller V Wikman H. Breast cancer brain metastases: biology and new clinical perspectives. Breast Cancer Res (2016) 18:8. doi: 10.1186/s13058-015-0665-1 26781299 PMC4717619

[3] Li XX Yang J Peng LM Sahin AA Huo L Ward KC. Triple-negative breast cancer has worse overall survival and cause-specific survival than non-triple-negative breast cancer. Breast Cancer Res Treat (2017) 161:279–87. doi: 10.1007/s10549-016-4059-6 27888421

[4] Witzel I Laakmann E Weide R Neunhoffer T Park-Simon TJ Schmidt M. Treatment and outcomes of patients in the brain metastases in breast cancer network registry. Eur J Cancer (2018) 102:1–9. doi: 10.1016/j.ejca.2018.07.004 30099223

[5] Gennari A Andre F Barrios CH Cortes J de Azambuja E DeMichele A. ESMO clinical practice guideline for the diagnosis, staging and treatment of patients with metastatic breast cancer. Ann Oncol (2021) 32:1475–95. doi: 10.1016/j.annonc.2021.09.019 34678411

[6] Hambrecht A Jandial R Neman J. Emerging role of brain metastases in the prognosis of breast cancer patients. Breast Cancer (2011) 3:79–91. doi: 10.2147/BCTT.S19967 24367178 PMC3846823

[7] Iida M Harari PM Wheeler DL Toulany M. Targeting AKT/PKB to improve treatment outcomes for solid tumors. Mutat Res (2020) 819-820:111690. doi: 10.1016/j.mrfmmm.2020.111690 32120136 PMC7169978

[8] Manning BD Toker A. AKT/PKB signaling: navigating the network. Cell (2017) 169:381–405. doi: 10.1016/j.cell.2017.04.001 28431241 PMC5546324

[9] Hinz N Jucker M. Distinct functions of AKT isoforms in breast cancer: a comprehensive review. Cell Commun Signal: CCS (2019) 17:154. doi: 10.1186/s12964-019-0450-3 31752925 PMC6873690

[10] Chin YR Toker A. Akt isoform-specific signaling in breast cancer: uncovering an anti-migratory role for palladin. Cell Adh Migr (2011) 5:211–4. doi: 10.4161/cam.5.3.15790 PMC3210203 21519185

[11] Dillon RL Marcotte R Hennessy BT Woodgett JR Mills GB Muller WJ. Akt1 and akt2 play distinct roles in the initiation and metastatic phases of mammary tumor progression. Cancer Res (2009) 69:5057–64. doi: 10.1158/0008-5472.CAN-08-4287 PMC4151524 19491266

[12] Ellis MJ Perou CM. The genomic landscape of breast cancer as a therapeutic roadmap. Cancer Discov (2013) 3:27–34. doi: 10.1158/2159-8290.Cd-12-0462 23319768 PMC3553590

[13] Ellis H Ma CX. PI3K inhibitors in breast cancer therapy. Curr Oncol Rep (2019) 21:110. doi: 10.1007/s11912-019-0846-7 31828441

[14] Sweeney C Bracarda S Sternberg CN Chi KN Olmos D Sandhu S. Ipatasertib plus abiraterone and prednisolone in metastatic castration-resistant prostate cancer (IPATential150): a multicentre, randomised, double-blind, phase 3 trial. Lancet (2021) 398:131–42. doi: 10.1016/S0140-6736(21)00580-8 34246347

[15] Turner N Dent RA O’Shaughnessy J Kim SB Isakoff SJ Barrios C. Ipatasertib plus paclitaxel for PIK3CA/AKT1/PTEN-altered hormone receptor-positive HER2-negative advanced breast cancer: primary results from cohort b of the IPATunity130 randomized phase 3 trial. Breast Cancer Res Treat (2022) 191:565–76. doi: 10.1007/s10549-021-06450-x PMC8831286 34860318

[16] Kim SB Dent R Im SA Espie M Blau S Tan AR. Ipatasertib plus paclitaxel versus placebo plus paclitaxel as first-line therapy for metastatic triple-negative breast cancer (LOTUS): a multicentre, randomised, double-blind, placebo-controlled, phase 2 trial. Lancet Oncol (2017) 18:1360–72. doi: 10.1016/S1470-2045(17)30450-3 PMC5626630 28800861

[17] Hohensee I Chuang HN Grottke A Werner S Schulte A Horn S. PTEN mediates the cross talk between breast and glial cells in brain metastases leading to rapid disease progression. Oncotarget (2017) 8:6155–68. doi: 10.18632/oncotarget.14047 PMC5351620 28008153

[18] Zhang L Zhang S Yao J Lowery FJ Zhang Q Huang WC. Microenvironment-induced PTEN loss by exosomal microRNA primes brain metastasis outgrowth. Nature (2015) 527:100–4. doi: 10.1038/nature15376 PMC4819404 26479035

[19] Sahlberg SH Gustafsson AS Pendekanti PN Glimelius B Stenerlow B. The influence of AKT isoforms on radiation sensitivity and DNA repair in colon cancer cell lines. Tumour Biol (2014) 35:3525–34. doi: 10.1007/s13277-013-1465-9 PMC3980041 24338765

[20] Ran FA Hsu PD Wright J Agarwala V Scott DA Zhang F. Genome engineering using the CRISPR-Cas9 system. Nat Protoc (2013) 8:2281–308. doi: 10.1038/nprot.2013.143 PMC3969860 24157548

[21] Dehairs J Talebi A Cherifi Y Swinnen JV. CRISP-ID: decoding CRISPR mediated indels by Sanger sequencing. Sci Rep-Uk (2016) 6:28973. doi: 10.1038/srep28973 PMC4929496 27363488

[22] Hamester F Legler K Wichert B Kelle N Eylmann K Rossberg M. Prognostic relevance of the golgi mannosidase MAN1A1 in ovarian cancer: impact of n-glycosylation on tumour cell aggregation. Br J Cancer (2019) 121:944–53. doi: 10.1038/s41416-019-0607-2 PMC6889143 31659304

[23] Meyer F Engel AM Krause AK Wagner T Poole L Dubrovska A. Efficient DNA repair mitigates replication stress resulting in less immunogenic cytosolic DNA in radioresistant breast cancer stem cells. Front Immunol (2022) 13:765284. doi: 10.3389/fimmu.2022.765284 35280989 PMC8913591

[24] Bellacosa A Testa JR Staal SP Tsichlis PN. A retroviral oncogene, akt, encoding a serine-threonine kinase containing an SH2-like region. Science (1991) 254:274–7. doi: 10.1126/science.254.5029.274 1833819

[25] Adamo B Deal AM Burrows E Geradts J Hamilton E Blackwell KL. Phosphatidylinositol 3-kinase pathway activation in breast cancer brain metastases. Breast Cancer Res (2011) 13:R125. doi: 10.1186/bcr3071 22132754 PMC3326567

[26] Saunus JM Quinn MCJ Patch AM Pearson JV Bailey PJ Nones K. Integrated genomic and transcriptomic analysis of human brain metastases identifies alterations of potential clinical significance. J Pathol (2015) 237:363–78. doi: 10.1002/path.4583 26172396

[27] Martorana F Motta G Pavone G Motta L Stella S Vitale SR. AKT inhibitors: new weapons in the fight against breast cancer? Front Pharmacol (2021) 12:662232. doi: 10.3389/fphar.2021.662232 33995085 PMC8118639

[28] Andre F Ciruelos EM Juric D Loibl S Campone M Mayer IA. Alpelisib plus fulvestrant for PIK3CA-mutated, hormone receptor-positive, human epidermal growth factor receptor-2-negative advanced breast cancer: final overall survival results from SOLAR-1. Ann Oncol (2021) 32:208–17. doi: 10.1016/j.annonc.2020.11.011 33246021

[29] Baselga J Campone M Piccart M Burris HA 3rd Rugo HS Sahmoud T. Everolimus in postmenopausal hormone-receptor-positive advanced breast cancer. N Engl J Med (2012) 366:520–9. doi: 10.1056/NEJMoa1109653 PMC5705195 22149876

[30] Cocco S Leone A Roca MS Lombardi R Piezzo M Caputo R. Inhibition of autophagy by chloroquine prevents resistance to PI3K/AKT inhibitors and potentiates their antitumor effect in combination with paclitaxel in triple negative breast cancer models. J Transl Med (2022) 20:290. doi: 10.1186/s12967-022-03462-z 35761360 PMC9235112

[31] Ippen FM Grosch JK Subramanian M Kuter BM Liederer BM Plise EG. Targeting the PI3K/Akt/mTOR pathway with the pan-akt inhibitor GDC-0068 in PIK3CA-mutant breast cancer brain metastases. Neuro Oncol (2019) 21:1401–11. doi: 10.1093/neuonc/noz105 PMC6827829 31173106

[32] Morgillo F Della Corte CM Diana A Mauro CD Ciaramella V Barra G. Phosphatidylinositol 3-kinase (PI3Kalpha)/AKT axis blockade with taselisib or ipatasertib enhances the efficacy of anti-microtubule drugs in human breast cancer cells. Oncotarget (2017) 8:76479–91. doi: 10.18632/oncotarget.20385 PMC5652721 29100327

[33] Lin J Sampath D Nannini MA Lee BB Degtyarev M Oeh J. Targeting activated akt with GDC-0068, a novel selective akt inhibitor that is efficacious in multiple tumor models. Clin Cancer Res (2013) 19:1760–72. doi: 10.1158/1078-0432.Ccr-12-3072 23287563

[34] Lin K Lin J Wu WI Ballard J Lee BB Gloor SL. An ATP-site on-off switch that restricts phosphatase accessibility of akt. Sci Signal (2012) 5:ra37. doi: 10.1126/scisignal.2002618 22569334

[35] Han EK Leverson JD McGonigal T Shah OJ Woods KW Hunter T. Akt inhibitor a-443654 induces rapid akt ser-473 phosphorylation independent of mTORC1 inhibition. Oncogene (2007) 26:5655–61. doi: 10.1038/sj.onc.1210343 17334390

[36] Mundi PS Sachdev J McCourt C Kalinsky K. AKT in cancer: new molecular insights and advances in drug development. Br J Clin Pharmacol (2016) 82:943–56. doi: 10.1111/bcp.13021 PMC5137819 27232857

[37] Rhodes N Heerding DA Duckett DR Eberwein DJ Knick VB Lansing TJ. Characterization of an akt kinase inhibitor with potent pharmacodynamic and antitumor activity. Cancer Res (2008) 68:2366–74. doi: 10.1158/0008-5472.CAN-07-5783 18381444

[38] Saura C Roda D Rosello S Oliveira M Macarulla T Perez-Fidalgo JA. A first-in-Human phase I study of the ATP-competitive AKT inhibitor ipatasertib demonstrates robust and safe targeting of AKT in patients with solid tumors. Cancer Discov (2017) 7:102–13. doi: 10.1158/2159-8290.CD-16-0512 PMC5463454 27872130

[39] Slotkin EK Diolaiti D Shukla NN Dela Cruz FS Clark JJ Gundem G. Patient-driven discovery, therapeutic targeting, and post-clinical validation of a novel AKT1 fusion-driven cancer. Cancer Discov (2019) 9:605–16. doi: 10.1158/2159-8290.CD-18-0953 PMC6497560 30877085

[40] Feng X Jiang J Shi S Xie H Zhou L Zheng S. Knockdown of miR-25 increases the sensitivity of liver cancer stem cells to TRAIL-induced apoptosis via PTEN/PI3K/Akt/Bad signaling pathway. Int J Oncol (2016) 49:2600–10. doi: 10.3892/ijo.2016.3751 27840896

[41] Garofalo M Di Leva G Romano G Nuovo G Suh SS Ngankeu A. miR-221&222 regulate TRAIL resistance and enhance tumorigenicity through PTEN and TIMP3 downregulation. Cancer Cell (2009) 16:498–509. doi: 10.1016/j.ccr.2009.10.014 19962668 PMC2796583

[42] Kim KH Kim HS Kim SC Kim D Kim YB Chung HC. Gene expression profiling identifies akt as a target for radiosensitization in gastric cancer cells. Front Oncol (2020) 10:562284. doi: 10.3389/fonc.2020.562284 33042843 PMC7517358

[43] Shimura T Kakuda S Ochiai Y Nakagawa H Kuwahara Y Takai Y. Acquired radioresistance of human tumor cells by DNA-PK/AKT/GSK3beta-mediated cyclin D1 overexpression. Oncogene (2010) 29:4826–37. doi: 10.1038/onc.2010.238 20562919

[44] Narayan RS Fedrigo CA Brands E Dik R Stalpers LJA Baumert BG. The allosteric AKT inhibitor MK2206 shows a synergistic interaction with chemotherapy and radiotherapy in glioblastoma spheroid cultures. BMC Cancer (2017) 17:204. doi: 10.1186/s12885-017-3193-9 PMC5359921 28320338

[45] Shimura T Kakuda S Ochiai Y Kuwahara Y Takai Y Fukumoto M. Targeting the AKT/GSK3beta/cyclin D1/Cdk4 survival signaling pathway for eradication of tumor radioresistance acquired by fractionated radiotherapy. Int J Radiat Oncol Biol Phys (2011) 80:540–8. doi: 10.1016/j.ijrobp.2010.12.065 21398050

[46] Lehman HL Van Laere SJ van Golen CM Vermeulen PB Dirix LY van Golen KL. Regulation of inflammatory breast cancer cell invasion through Akt1/PKB alpha phosphorylation of RhoC GTPase. Mol Cancer Res (2012) 10:1306–18. doi: 10.1158/1541-7786.Mcr-12-0173 22896661

[47] Chang TY Chen HA Chiu CF Chang YW Kuo TC Tseng PC. Dicer elicits paclitaxel chemosensitization and suppresses cancer stemness in breast cancer by repressing AXL. Cancer Res (2016) 76:3916–28. doi: 10.1158/0008-5472.CAN-15-2555 27216190

[48] Nassar A Abouelhoda M Mansour O Loutfy SA Hafez MM Gomaa M. Targeted next generation sequencing identifies somatic mutations in a cohort of Egyptian breast cancer patients. J Adv Res (2020) 24:149–57. doi: 10.1016/j.jare.2020.04.001 PMC7167517 32322420

[49] Wang L Man N Sun XJ Tan Y Garcia-Cao M Liu F. Regulation of AKT signaling by Id1 controls t(8;21) leukemia initiation and progression. Blood (2015) 126:640–50. doi: 10.1182/blood-2015-03-635532 PMC4520879 26084673

[50] Toska E Osmanbeyoglu HU Castel P Chan C Hendrickson RC Elkabets M. PI3K pathway regulates ER-dependent transcription in breast cancer through the epigenetic regulator KMT2D. Science (2017) 355:1324–30. doi: 10.1126/science.aah6893 PMC5485411 28336670

