# Supplementary figures and images for: Impact of AKT1 on cell invasion and radiosensitivity in a triple negative breast cancer cell line developing brain metastasis
Source: Front Oncol. 2023 Jul 6;13:1129682. doi: 10.3389/fonc.2023.1129682 (PMC10358765; doi:10.3389/fonc.2023.1129682)

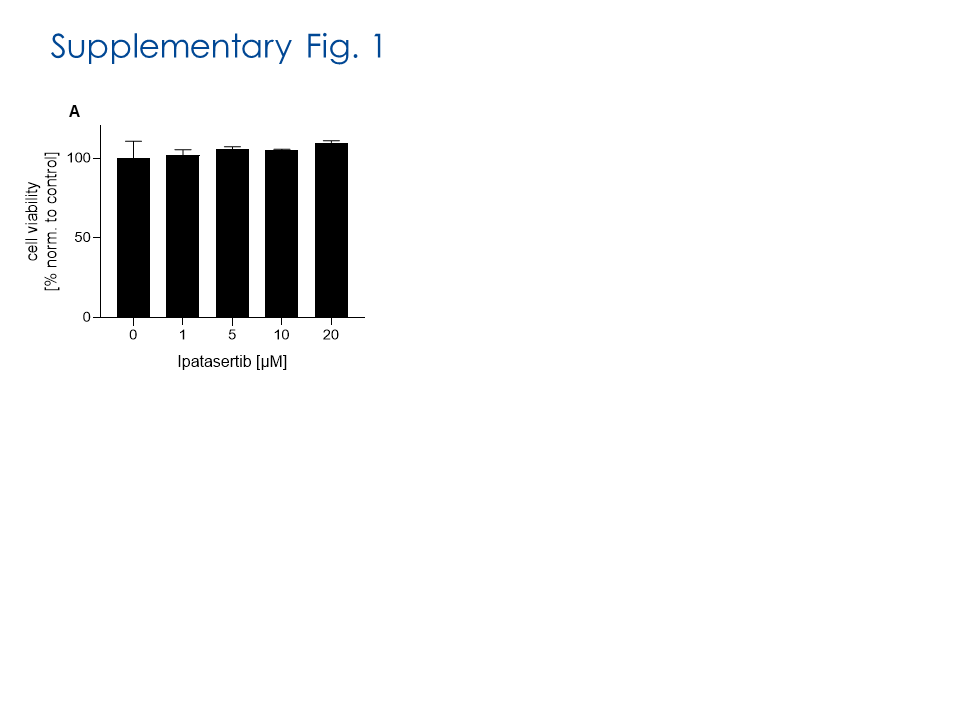

Supplement: Supplementary Figure 1 — Influence of Ipatasertib on proliferation of MDA-MB-231 breast cancer cells. Proliferation of MDA-MB-231 cells in serum reduced growth medium after treatment with 1, 5, 10 and 20µM Ipatasertib. Cell viability was determined by WST-1 reagent after 72h. Data are presented as mean ± S.D.. Statistic was assessed by one-way ANOVA with Bonferroni post hoc tests with the result that p > 0.05 is considered as non-significant. [file Image_1.tif]

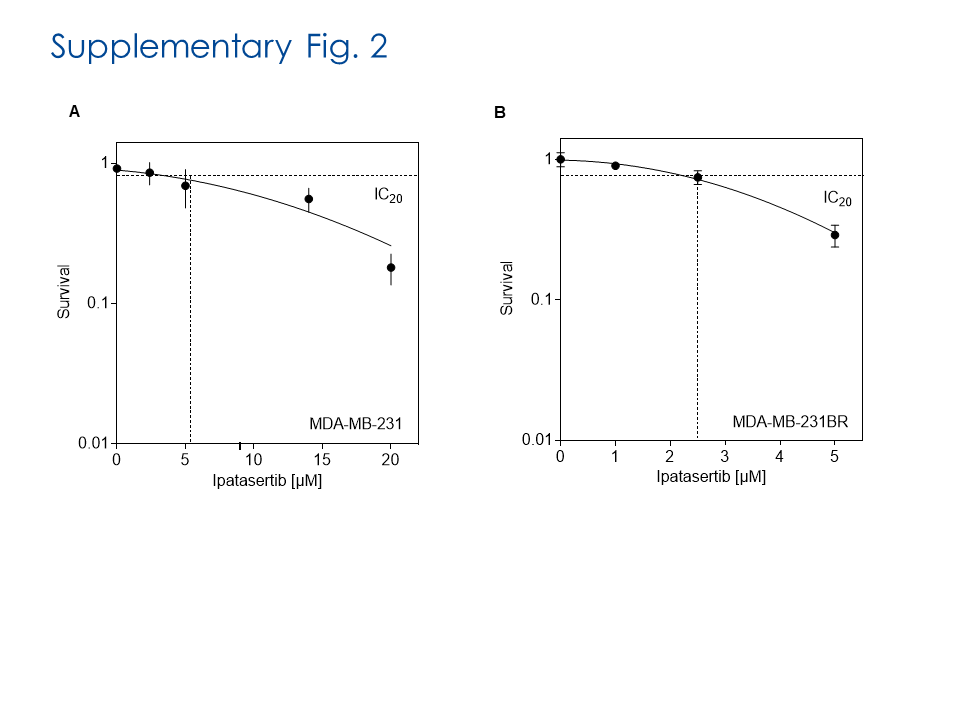

Supplement: Supplementary Figure 2 — Influence of Ipatasertib on radiation in MDA-MB-231BR breast cancer cells. (A) MDA-MB-231 cells were treated with Ipatasertib and were radiated with 2, 4 and 6Gy. To estimate IC20-value, colony formation was determined after 10d. (B) MDA-MB-231BR cells were treated with Ipatasertib and were radiated with 2, 4 and 6Gy. To estimate IC20-value, colony formation was determined after 10d. Data are presented as mean ± S.D. * = p<0.05; ** = p<0.01; *** = p<0.001 as assessed by one-way ANOVA with Bonferroni post hoc tests or by two-way ANOVA with Bonferroni post hoc tests. If not stated otherwise, p>0.05 is considered non-significant. [file Image_2.tif]
