# Supplementary material for: Impact of AKT1 on cell invasion and radiosensitivity in a triple negative breast cancer cell line developing brain metastasis
Source: Front Oncol. 2023 Jul 6;13:1129682. doi: 10.3389/fonc.2023.1129682 (PMC10358765; doi:10.3389/fonc.2023.1129682)
Supplement: Supplementary file 6 [file DataSheet_1.docx]

Supplementary Materials and Methods part:

**CRISPR/Cas-9 knockout of AKT1 in MDA-MB-231BR**

guide RNA’s for AKT1 knockout (KO), generated with the CRISPR guide RNA design tool *guide scan*:

**AKT1_hs_exon3**

**guide #1** (score:87, off-targets:0, Exon3, genomic coordinates: chr14: 104763101-104763146)

guide sequence: 5´ CACC GAGCGACGTGGCTATTGTGA 3´

complementary: 3´ CTCGCTGCACCGATAACACT CAAA 5’

guide #1_fwd: 5’ CACC GAGCGACGTGGCTATTGTGA 3’
guide #1_rev: 5’ AAAC TCACAATAGCCACGTCGCTC 3’

**AKT1_hs_exon4**

**guide #5** (score:85, off-targets:0, Exon4, genomic coordinates: chr14:104715528-104715656)

guide sequence: 5´ CACC TGGCTACAAGGAGCGGCCGC 3´
complementary: 3´ ACCGATGTTCCTCGCCGGCG CAAA 5’

guide #5_fwd: 5’ CACC TGGCTACAAGGAGCGGCCGC 3’

guide #1_rev: 5’ AAAC GCGGCCGCTCCTTGTAGCCA 3’

*WGS analysis*

Sequence data analysis was carried out by the UKE Bioinformatic Core Facility. Therefore, Fastp (v0.20.1) was used to remove artificial and low-quality (Phred quality score below 15) sequences from the 3′-end of sequence reads [1]. The reads were then aligned to the human reference assembly (GRCh38) using the Burrows Wheeler Aligner (BWA mem, v0.7.17-r1188) [2]. Putative PCR duplicates were removed with Samtools (v 1.10) [3]. Variant calling was performed using DeepVariant (v1.3.0) and variants were annotated with the Ensembl Variant Effect Predictor (VEP, cache v105) [4, 5].

*RNAseq analysis*

Sequence data analysis was carried out by the UKE Bioinformatic Core Facility. First, Fastp (v0.20.1) was used to remove artificial and low-quality (Phred quality score below 15) sequences from the 3′-end of sequence reads. Sequence reads were aligned to the human reference assembly (GRCh38.104) with STAR (v.2.7.9a) [6]. Normalization and differential expression analysis were carried out with DESeq2 [7]. Genes were considered to be differentially expressed if an absolute log2 fold change≥1 and an FDR≤0.1 were observed. Over-representation and gene set enrichment analyses were carried out with clusterProfiler [8].

References:

1. Chen S, Zhou Y, Chen Y & Gu J (2018) fastp: an ultra-fast all-in-one FASTQ preprocessor. Bioinformatics 34, i884-i890, doi: 10.1093/bioinformatics/bty560.

2. Li H & Durbin R (2009) Fast and accurate short read alignment with Burrows-Wheeler transform. Bioinformatics 25, 1754-1760, doi: 10.1093/bioinformatics/btp324.

3. Li H, Handsaker B, Wysoker A, Fennell T, Ruan J, Homer N, Marth G, Abecasis G, Durbin R & Genome Project Data Processing S (2009) The Sequence Alignment/Map format and SAMtools. Bioinformatics 25, 2078-2079, doi: 10.1093/bioinformatics/btp352.

4. McLaren W, Gil L, Hunt SE, Riat HS, Ritchie GR, Thormann A, Flicek P & Cunningham F (2016) The Ensembl Variant Effect Predictor. Genome Biol 17, 122, doi: 10.1186/s13059-016-0974-4.

5. Poplin R, Chang PC, Alexander D, Schwartz S, Colthurst T, Ku A, Newburger D, Dijamco J, Nguyen N, Afshar PT, Gross SS, Dorfman L, McLean CY & DePristo MA (2018) A universal SNP and small-indel variant caller using deep neural networks. Nat Biotechnol 36, 983-987, doi: 10.1038/nbt.4235.

6. Dobin A, Davis CA, Schlesinger F, Drenkow J, Zaleski C, Jha S, Batut P, Chaisson M & Gingeras TR (2013) STAR: ultrafast universal RNA-seq aligner. Bioinformatics 29, 15-21, doi: 10.1093/bioinformatics/bts635.

7. Love MI, Huber W & Anders S (2014) Moderated estimation of fold change and dispersion for RNA-seq data with DESeq2. Genome Biol 15, 550, doi: 10.1186/s13059-014-0550-8.

8. Wu T, Hu E, Xu S, Chen M, Guo P, Dai Z, Feng T, Zhou L, Tang W, Zhan L, Fu X, Liu S, Bo X & Yu G (2021) clusterProfiler 4.0: A universal enrichment tool for interpreting omics data. Innovation (Camb) 2, 100141, doi: 10.1016/j.xinn.2021.100141.
